# Association between metabolic syndrome, C-reactive protein, and the risk of primary liver cancer: a large prospective study

**DOI:** 10.1186/s12885-022-09939-w

**Published:** 2022-08-04

**Authors:** Mengmeng Song, Tong Liu, Hai Liu, Qi Zhang, Qingsong Zhang, Yiming Wang, Xiangming Ma, Liying Cao, Hanping Shi

**Affiliations:** 1 Department of Gastrointestinal Surgery/Clinical Nutrition, Beijing Shijitan Hospital, Capital Medical University, Beijing, 100038 China; 2 Key Laboratory of Cancer FSMP for State Market Regulation, Beijing, 100038 China; 3 Beijing International Science and Technology Cooperation Base for Cancer Metabolism and Nutrition, Beijing, 100038 China; 4 Department of Anesthesia, Kailuan General Hospital, Tangshan, China; 5 Department of General Surgery, Kailuan General Hospital, Tangshan, 063000 China; 6 Department of Hepatological Surgery, Kailuan General Hospital, Tangshan, 063000 China

**Keywords:** Primary liver cancer, Metabolic syndrome, Incidence, Hs-CRP

## Abstract

**Background and aims:**

High-sensitivity C-reactive protein (hs-CRP) levels and metabolic syndrome (MetS) are known to be associated with an increased incidence of different cancers. We aimed to evaluate the effect of MetS combined with high hs-CRP levels on the risk of primary liver cancer (PLC).

**Methods:**

Participants were recruited from the Kailuan cohort study and were classified into four groups according to the presence or absence of MetS and inflammation (hs-CRP ≥ 3 or < 3 mg/L). The associations of MetS and inflammation with the risk of PLC were assessed using Cox proportional hazards models.

**Results:**

This study included 92,770 participants. The mean age was 51.4 years old. Over a median follow-up of 13.02 years, 395 participants were diagnosed as PLC. Compared to the control participants without inflammation (hs-CRP < 3 mg/L) and MetS (*n* = 69,413), participants with high hs-CRP levels combined with MetS (*n* = 2,269) had a higher risk of PLC [hazard ratios (HR) 2.91; 95% confidence interval (CI), 1.77–4.81], and participants with high hs-CRP levels and without MetS (*n* = 14,576) had the same trend (HR, 1.36; 95%CI, 1.05–1.75). However, participants with low hs-CRP levels and MetS (*n* = 6,512) had no significant association with an elevated risk of PLC (HR, 1.18; 95%CI, 0.76–1.82). After excluding participants who had cancer during the first year of follow-up, sensitivity analysis showed the same trend. In addition, co-occurrence of MetS and high hs-CRP levels had significant interactive effects on the risk of PLC between the sexes (*P* < 0.001) and the patients with HBV infection (*P* = 0.012).

**Conclusions:**

Participants with co-occurrence of MetS and high hs-CRP levels have an elevated risk of PLC.

**Trial registration:**

Kailuan study, ChiCTR–TNRC–11001489. Registered 24 August, 2011-Retrospectively registered, http://www.chictr.org.cn/showprojen.aspx?proj=8050

**Supplementary Information:**

The online version contains supplementary material available at 10.1186/s12885-022-09939-w.

## Lay summary

High levels of inflammation and the prevalence of metabolic syndrome are known to be associated with an increased risk for various cancers. Through this large prospective cohort study, we found that the co-occurrence of MetS and high hs-CRP levels is associated with an increased risk of new-onset PLC in the Chinese population. Thus, co-occurrence of MetS and high hs-CRP can help recognize the population high risk of PLC. More in-depth physical examination and liver cancer screening for this population is conducive to early detection, early intervention and early treatment of PLC.

## Introduction

Primary liver cancer (PLC) is the sixth most frequently diagnosed cancer worldwide and the third most common cause of cancer-related mortality [1]. China is a high-risk area for liver cancer. The recognized main risk factors for PLC include chronic infection with hepatitis B virus (HBV) or hepatitis C virus (HCV), alcoholic liver disease, aflatoxin-contaminated foods, excess body weight, type 2 diabetes (T2DM), and smoking. Importantly, there is a transition appearing in the major risk factors of liver cancer in recent years, with the prevalence of HBV and HCV declining and metabolic conditions (excess body weight and diabetes) increasing [2].

Increasing evidence suggests that metabolic conditions, such as obesity [3], and diabetes [4] are associated with an increased risk of PLC. Metabolic syndrome (MetS), a cluster of metabolic disorders, including high blood pressure, diabetes, high triglyceride, central obesity and low high-density lipoprotein, is reported to have a high prevalence (between 10%-30% in the adult population) in both the developed and developing countries [5]. Accumulating evidence from epidemiological studies indicates that MetS has been linked to an increased risk of the development of chronic diseases [6] and cancer, such as PLC [7]. Importantly, recent studies found that nonalcoholic fatty liver disease (NAFLD) was the underlying cause of 13–38.2% of patients with liver cancer unrelated with viruses or alcohol [8]. Although approximately 85% of hepatocellular carcinomas occur within a background of liver cirrhosis, a significant number of cases of NAFLD-associated liver cancer occur in non-cirrhotic livers, especially in patients with multiple metabolic risk factors [2]. A large European study also found that NAFLD was present in 94% of obese patients, including 25% of nonalcoholic steatohepatitis (NASH), patients, and the overall prevalence of NAFLD in patients with T2DM was 40%-70% [9]. Thus, NAFLD is tightly associated with the metabolic syndrome (MetS), and the link between MeTS and liver cancer is likely mediated through the NAFLD pathway.

In addition, inflammation, as the hallmark of PLC, has been reported to be associated with an increased risk of liver cancer in several studies [10, 11]. Our previous study has shown that the level of high-sensitivity C-reactive protein (hs-CRP), a sensitive indicator of inflammatory status, is associated with an increased risk of PLC [12]. Inflammation, as a systemic response, has a complex crosstalk relationship with MetS. Particularly, the inflammation associated with MetS has distinct manifestations [13]. The dimension of inflammation activation is not large, and it is often referred to as “low-grade” inflammation or “metaflammation” [14], which is inflammation that is caused by metabolism. Therefore, we hypothesized that the combination of MetS and high hs-CRP levels may be associated with a higher risk of incident PLC. We conducted a prospective, population-based cohort study to assess whether the co-occurrence of MetS and high hs-CRP levels is associated with the elevated risk of PLC.

## Methods

### Study design and population

**Fig. 1:**
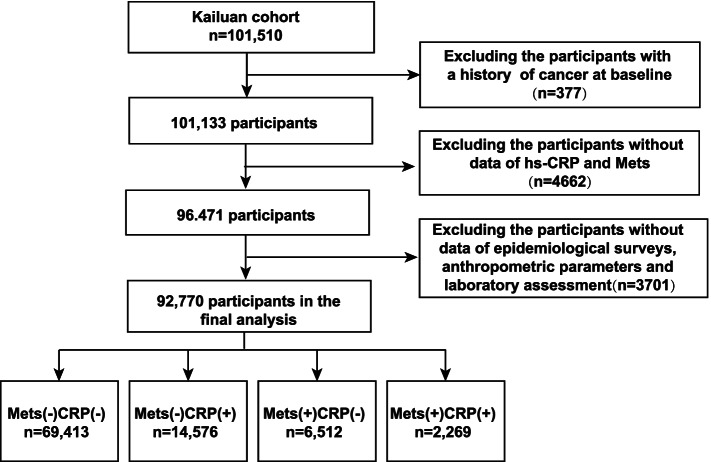
Flow chart of study participants. Hs-CRP, high sensitivity C-reactive protein; MetS, metabolic syndrome

The Kailuan Study (Registration number: ChiCTR–TNRC–11001489; http://www.chictr.org.cn/showprojen.aspx?proj=8050; The date of first registration: 24/08/2011) is a large prospective cohort study conducted in the Kailuan community (Tangshan City, Hebei Province, China), and it aims to explore the risk factors of chronic diseases. The design and methodology of the study have been described in detail in a previous study [15]. In total, 101,510 participants (aged 18–98 years; 20,400 women and 81,110 men) were enrolled in the Kailuan study, and the first examination was performed between June 2006 and October 2007. Subsequently, the participants were followed up every 2 years and assessed using standardized questionnaires, clinical examinations, and laboratory tests. A flowchart of the present study is shown in Fig. 1. We excluded participant with a history of cancer (*n* = 377), and without baseline data for metabolic syndrome diagnosis or hs-CRP (*n* = 4,662). In addition, 3,701 participants of all subjects lacking laboratory examination, epidemiological surveys or anthropometric parameters were also excluded. Finally, 92,770 participants were enrolled in this study. The study protocol conformed to the ethical guidelines of the 1975 Declaration of Helsinki and was approved by the ethics committees of Kailuan General Hospital and Beijing Shijitan Hospital. Signed informed consent forms was obtained from all the participants.

### Collection and definitions of variables

All baseline data were obtained from participants who attended their first physical examination (2006–2007). Waist circumference (WC) was measured with a tape measure using the midpoint of the line between the lower edge of the rib and the upper edge of the hip as a reference point. Blood pressure (BP) was continuously measured twice using a mercury sphygmomanometer with the participant in an upright seated position after resting for 5 min, and the average BP was taken for subsequent analysis. Hypertension was defined as systolic blood pressure (SBP) ≥ 140 mmHg, diastolic blood pressure (DBP) ≥ 90 mmHg, current treatment with antihypertensive medication or self-reported history of hypertension. Fasting blood (8–12 h of fasting) specimens of blood were collected, and fasting plasma glucose (FPG) concentration was determined using the hexokinase/glucose-6-phosphate dehydrogenase method. Diabetes mellitus (DM) was defined as FPG ≥ 7.0 mmol/L, random plasma glucose ≥ 11.1 mmol/L, or self-reported diabetes history or current treatment with anti-diabetic medication. Triglyceride levels were determined using the enzyme colorimetric method, whereas the high-density lipoprotein cholesterol (HDL-C) concentration was measured using the direct method. Serum hs-CRP levels were assessed using a high-sensitivity immunoturbidimetric method (Cias Latex CRP-H, Kanto Chemical Co. Inc, Tokyo, Japan) with a detection limit of 0.1 mg/L. High hs-CRP levels were defined as serum hs-CRP levels > 3 mg/L according to the Centers for Disease Control and Prevention and the American Heart Association guidelines [16].

### Definition of MetS and the subgroups

According to common definition of MetS in 2009 [17], MetS is defined as having the following three or more parameters: 1) FPG > 5.6 mmol/L, or have received appropriate treatment, 2) Systolic blood pressure ≥ 130 mmHg and/or diastolic ≥ 85 mmHg, or have received appropriate treatment, 3) Triglycerides ≥ 1.69 mmol/L, or have received appropriate treatment, 4) High-density lipoprotein in males < 1.04 mmol/L or < 1.29 mmol/L in females, or have received appropriate treatment, and 5) Obesity: WC ≥ 85 cm in men or ≥ 80 cm in women (cutoff points of WC for China).

The participants were divided into four groups according to the presence or absence of MetS and the levels of hs-CRP (hs-CRP ≤ 3 mg/L or > 3 mg/L): 1) MetS-CRP-, participants without MetS and with hs-CRP levels ≤ 3 mg/L, 2) MetS-CRP + , participants without MetS and with hs-CRP levels > 3 mg/L, 3) MetS + CRP-, participants with MetS and with hs-CRP levels ≤ 3 mg/L, and 4) MetS + CRP + , participants with MetS and hs-CRP levels > 3 mg/L. In addition, we systematically evaluated the number of MetS components [ranging from 0 (no positive syndromes) to 5 (all positive syndromes)] to assess the dose–response relationship between the degree of metabolic disorders and the risk of PLC. Due to the limited number of cases, patients with four and five components of MetS were merged into one group.

### Definition of study outcomes

The outcome of our study was the occurrence of PLC, which was identified by the following sources: 1) clinical examination conducted every 2 years until December 31, 2019, 2) medical records of Tangshan Medical Insurance System and Kailuan Social Insurance Information System, and 3) death certificates from the Provincial Vital Statistics Office (PVSO) to obtain additional missing information. Clinical experts assessed the diagnosis and classified patients PLC into C22 according to the 10th edition of the International Classification of Diseases.

### Statistical analysis

Normally distributed variables were expressed as mean ± standard deviation (SD), and comparisons between groups were performed using one-way analysis of variance. The median (interquartile range, IQR) was used to describe the non-normally distributed variables (hs-CRP and triglyceride levels), and nonparametric tests were used for comparison. Absolute values (percentages) were used to describe categorical variables, and the chi-square test was used for comparison. The person-year was calculated from the date of baseline inspection to the date of PLC diagnosis, the date of death, or December 31, 2019 (whichever occurred first). Logistic regression was used to calculate odds ratios (ORs) to estimate the association between MetS components and inflammation. The Cox proportional hazard model was used to calculate hazard ratios (HRs), and 95% confidence intervals (CIs) to estimate the impact of MetS and inflammation (hs-CRP > 3 mg/L) alone and in combination on the risk of PLC. The time variable used to create the survival time dataset was follow-up time. Adjusted factors included age (10-year age classes), sex, family income, educational background, marital status, body mass index (BMI), total cholesterol, alanine transaminase (ALT), serum uric acid (SUA), smoking status, drinking status, physical activity, sedentary lifestyle, tea consumption, salt intake, high-fat diet, and family history of cancer. The subgroup analysis stratified the participants by sex (male vs. female), and age (< 65 years vs. ≥ 65 years) and whether they had hepatitis virus infection. In the sensitivity analysis, we excluded participants who had cancer during the first year of follow-up to exclude the influence of patients who had underlying cancer but were not detected. The interactions between MetS/hs-CRP and these variables were further tested by multiplicative models. Statistical significance was defined as a two-sided *P* value < 0.05. A commercially available software program (SAS software, version 9.4) was used for statistical analysis.

## Results

### Baseline characteristics

A total of 92,770 participants were included in the study. The mean age was 51.48 years old. 74,132 (79.91%) were male. The median (IQR) of hs-CRP was 0.80 (IQR, 0.30–2.06). 8,781 (9.47%) participants had MetS, and 16,845 (17.77%) had a high hs-CRP levels. Among all participants, 2,567 (2.77%) were HBsAg seropositive. The details were shown in Table 1.

**Table 1:** Baseline characteristics of the participants stratified by MetS and hs-CRP status

Variables	Overall	MetS-CRP-	MetS-CRP +	MetS + CRP-	MetS + CRP +	*P*-value
N	92,770	69,413	14,576	6,512	2,269	
Age (year)	51.48 ± 12.44	50.50 ± 12.46	54.77 ± 13.04	53.12 ± 9.69	55.61 ± 10.00	< 0.001
Hs-CRP (mg/L)	0.80(0.30,2.06)	0.55(0.22,1.13)	5.92(4.00,9.14)	0.83(0.38,1.55)	5.80(3.89,8.80)	< 0.001
WC (cm)	86.95 ± 9.97	85.88 ± 9.67	89.26 ± 10.56	90.72 ± 8.88	93.85 ± 9.86	< 0.001
FPG (mmol/L)	5.48 ± 1.68	5.32 ± 1.43	5.41 ± 1.76	6.84 ± 1.81	7.18 ± 1.92	< 0.001
SBP (mmHg)	131.08 ± 21.06	128.79 ± 20.06	131.94 ± 21.44	147.49 ± 20.09	148.40 ± 21.56	< 0.001
DBP (mmHg)	83.61 ± 11.78	82.50 ± 11.31	83.17 ± 11.73	93.28 ± 11.11	92.65 ± 11.40	< 0.001
HDL-C (mmol/L)	1.55 ± 0.40	1.55 ± 0.39	1.55 ± 0.41	1.51 ± 0.43	1.53 ± 0.47	< 0.001
TG (mmol/L)	1.27(0.90,1.93)	1.52(1.29,1.77)	1.50(1.28,1.76)	1.44(1.22,1.75)	1.46(1.23,1.76)	< 0.001
Male, n (%)	74,132(79.91)	57,299(82.55)	12,115(83.12)	3668(56.33)	1050(46.28)	< 0.001
Reported income (¥)						< 0.001
< 600	26,807(28.90)	20,607(29.69)	3850(26.41)	1828(28.07)	522(23.01)	
600–800	52,731(56.84)	38,815(55.92)	8668(59.47)	3822(58.69)	1426(62.85)	
800–1000	7096(7.65)	5315(7.66)	1129(7.75)	479(7.36)	173(7.62)	
> 1000	6136(6.61)	4676(6.74)	929(6.37)	383(5.88)	148(6.52)	
Marital status, n (%)						< 0.001
Never	1558(1.68)	1340(1.93)	193(1.32)	17(0.26)	8(0.35)	
Married	87,536(94.36)	65,568(94.46)	13,631(93.52)	6204(95.27)	2133(94.01)	
Divorced	794(0.86)	587(0.85)	126(0.86)	53(0.81)	28(1.23)	
Widowed	1913(2.06)	1219(1.76)	445(3.05)	168(2.58)	81(3.57)	
Remarried	969(1.04)	699(1.01)	181(1.24)	70(1.07)	19(0.84)	
Educational background, n (%)						< 0.001
Never	1136(1.22)	760(1.09)	286(1.96)	63(0.97)	27(1.19)	
Primary school	8935(9.63)	6282(9.05)	1768(12.13)	652(10.01)	233(10.27)	
Middle school	64,370(69.39)	48,195(69.43)	9761(66.97)	4781(73.42)	1633(71.97)	
High school	12,059(13.00)	9223(13.29)	1785(12.25)	772(11.86)	279(12.30)	
College graduate or above	6270(6.76)	4953(7.14)	976(6.70)	244(3.75)	97(4.28)	
TC, n (%)						< 0.001
< 4.51 mmol/L	31,011(33.43)	23,851(34.36)	5067(34.76)	1570(24.11)	523(23.05)	
4.51 ~ 5.34 mmol/L	31,039(33.46)	23,675(34.11)	4876(33.45)	1824(28.01)	664(29.26)	
> 5.34 mmol/L	30,720(33.11)	21,887(31.53)	4633(31.79)	3118(47.88)	1082(47.69)	
ALT, n (%)						< 0.001
< 14.90 u/L	30,918(33.33)	23,326(33.60)	5177(35.52)	1785(27.41)	630(27.77)	
14.90 ~ 22.00 u/L	32,410(34.94)	24,644(35.50)	4811(33.01)	2229(34.23)	726(32.00)	
> 22.00 u/L	29,442(31.74)	21,443(30.89)	4588(31.48)	2498(38.36)	913(40.24)	
UA, n (%)						< 0.001
< 249.40 μmol/L	30,893(33.30)	23,227(33.46)	4700(32.24)	2207(33.89)	759(33.45)	
249.40 ~ 317.00 μmol/L	31,165(33.59)	23,914(34.45)	4471(30.67)	2076(31.88)	704(31.03)	
> 317.00 μmol/L	30,712(33.11)	22,272(32.09)	5405(37.08)	2229(34.23)	806(35.52)	
BMI, n (%)						< 0.001
< 24 kg/m^2^	36,510(39.36)	29,715(42.81)	5371(36.85)	1142(17.54)	282(12.43)	
24–28 kg/m^2^	38,870(41.90)	28,764(41.44)	6004(41.19)	3081(47.31)	1021(45.00)	
> 28 kg/m^2^	17,390(18.75)	10,934(15.75)	3201(21.96)	2289(35.15)	966(42.57)	
Physical exercise, n (%)						< 0.001
Never	8093(8.72)	6345(9.14)	1117(7.66)	493(7.57)	138(6.08)	
Occasionally	70,112(75.58)	52,201(75.20)	11,306(77.57)	4834(74.23)	1771(78.05)	
Regularly	14,565(15.70)	10,867(15.66)	2153(14.77)	1185(18.20)	360(15.87)	
Smoking status, n (%)						< 0.001
Never	55,465(59.79)	40,262(58.00)	8844(60.68)	4596(70.58)	1763(77.70)	
Past	5282(5.69)	3921(5.65)	956(6.56)	314(4.82)	91(4.01)	
Moderate	3285(3.54)	2628(3.79)	439(3.01)	176(2.70)	42(1.85)	
Severe	28,738(30.98)	22,602(32.56)	4337(29.75)	1426(21.90)	373(16.44)	
Drinking status, n (%)						< 0.001
Never	54,718(58.98)	39,463(56.85)	8969(61.53)	4536(69.66)	1750(77.13)	
Past	3595(3.88)	2633(3.79)	712(4.88)	187(2.87)	63(2.78)	
Moderate	17,818(19.21)	14,235(20.51)	2545(17.46)	821(12.61)	217(9.56)	
Severe	16,639(17.94)	13,082(18.85)	2350(16.12)	968(14.86)	239(10.53)	
Sedentary lifestyle, n (%)						< 0.001
< 4 h/day	69,367(74.77)	51,491(74.18)	11,189(76.76)	4931(75.72)	1756(77.39)	
4–8 h/day	20,398(21.99)	15,625(22.51)	2960(20.31)	1386(21.28)	427(18.82)	
> 8 h/day	3005(3.24)	2297(3.31)	427(2.93)	195(2.99)	86(3.79)	
Tea consumption, n (%)						< 0.001
Never	69,689(75.12)	51,709(74.49)	11,045(75.78)	5116(78.56)	1819(80.17)	
< 1 time /month	4158(4.48)	3266(4.71)	606(4.16)	212(3.26)	74(3.26)	
-3 times/month	5604(6.04)	4271(6.15)	905(6.21)	330(5.07)	98(4.32)	
1–3 times/week	4582(4.94)	3555(5.12)	659(4.52)	288(4.42)	80(3.53)	
> 4 times/week	8737(9.42)	6612(9.53)	1361(9.34)	566(8.69)	198(8.73)	
High-fat diets, n (%)						< 0.001
Seldom	7844(8.46)	5954(8.58)	1133(7.77)	564(8.66)	193(8.51)	
Occasionally	76,344(82.29)	56,920(82.00)	12,201(83.71)	5315(81.62)	1908(84.09)	
Regularly	8582(9.25)	6539(9.42)	1242(8.52)	2326(6.79)	168(7.40)	
Salt intake, n (%)						< 0.001
Low (< 6 g/day)	8553(9.22)	6539(9.42)	1220(8.38)	605(9.29)	189(8.34)	
Intermediate (6–10 g/day)	74,161(79.98)	55,250(79.63)	11,828(81.23)	5226(80.29)	1857(81.95)	
High (> 10 g/day)	10,056(10.84)	7624(10.98)	1528(10.48)	681(10.46)	223(9.83)	
Family history of cancer, n (%)	3388(3.65)	2531(3.65)	545(3.74)	277(3.49)	85(3.75)	0.828
Diabetes mellitus, n (%)	7725(8.33)	3986(5.74)	1189(8.16)	1785(27.41)	765(33.72)	< 0.001
Hypertension, n (%)	38,296(41.28)	24,932(35.92)	6213(42.62)	5311(81.56)	1840(81.09)	< 0.001
HBsAg Seropositive, n (%)	2567(2.77)	2035(2.93)	363(2.49)	134(2.06)	35(1.54)	< 0.001
Fatty liver, n (%)	1777(1.92)	892(1.29)	360(2.47)	344(5.28)	181(7.98)	< 0.001
Liver cirrhosis, n (%)	158(0.17)	115(0.17)	39(0.27)	3(0.05)	1(0.04)	0.001

*hs-CRP* high-sensitivity C-reactive protein, *WC* Waist circumference, *FPG* Fasting plasma glucose, *SBP* Systolic blood pressure, *DBP* Diastolic blood pressure, *HDL-C* High-density lipoprotein cholesterol, *TG* Triglyceride, *BMI* Body mass index, *TC* Total cholesterol, *ALT* Alanine aminotransferase, *SUA* Serum uric acid

All participants were classified into four groups based on the presence or absence of MetS combined with the level of hs-CRP: MetS-CRP- group (*n* = 69,413), MetS-CRP + group (*n* = 14,576), MetS + CRP- group (*n* = 6,512), and MetS + CRP + group (*n* = 2,269). The baseline characteristics of the four groups are summarized in Table 1. The average age of the study population was 51.48 ± 12.44. The proportion of males in the MetS-CRP-, MetS-CRP + , MetS + CRP-, and MetS + CRP + groups was 82.55%, 83.12%, 56.33%, and 46.28%, respectively. Significant differences were found in age, levels of hs-CRP, WC, FPG, SBP, DBP, HDL-C, TG, sex, TC, ALT, UA, and BMI. In addition, other than family history of cancer, the percentages of reported income, marital status, educational background, physical activity, smoking status, drinking status, sedentary lifestyle, tea consumption, high-fat diets, salt intake, diabetes mellitus, hypertension, HBsAg seropositive, fatty liver and liver cirrhosis were different significantly between groups (all *P* < 0.05).

### Association of the relationship between MetS and hs-CRP levels with the incidence of PLC

The median follow-up time was 13.02 years (IQR, 12.70–13.20). By the end of the study, 395 participants had new-onset PLC. We performed univariate and multivariate Cox analyses to evaluate the association between MetS, hs-CRP levels, and the risk of PLC. The results are shown in Table 2, indicating that MetS was associated with a higher risk of PLC in participants with four or five MetS components (HR, 3.32; 95%CI, 1.46–7.56) than in participants without MetS. Compared with participants without MetS, those with MetS were associated with a 1.45-fold (HR, 1.45; 95%CI, 1.03–2.04) elevated risk of PLC. Moreover, elevated hs-CRP levels (> 3 mg/L) were also found to be associated with a higher risk of PLC (HR, 1.47; 95%CI, 1.17–1.86). However, there was no significant interaction between MetS and inflammation (hs-CRP > 3 mg/L) and the risk of PLC (*p* for interaction = 0.078).

**Table 2:** Hazard ratios (HRs) for the association of metabolic syndrome or hs-CRP levels with PLC risk

Group	Cases/ person-years	Crude models		Adjusted models	
		HR (95%CI)	*P*-value	HR (95%CI)	*P*-value
MetS metrics ^a^					
MetS-0	119/349088	Ref		Ref	
MetS-1	161/418573	1.12(0.88,1.42)	0.345	1.16(0.91,1.48)	0.229
MetS-2	74/261098	0.83(0.62,1.11)	0.211	0.89(0.66,1.21)	0.465
MetS-3	33/91177	1.06(0.72,1.56)	0.761	1.36(0.90,2.05)	0.141
MetS-4 (5)	8/15120	1.55(0.76,3.17)	0.231	3.32(1.46,7.56)	0.004
*P* for trend			0.197		0.010
MetS ^a^					
0	354/1028759	Ref		Ref	
1	41/106297	1.12(0.81,1.55)	0.479	1.45(1.03,2.04)	0.034
*P* for interaction ^b^					0.078
Hs-CRP ^c^					
≤ 3 mg/L	299/936212	Ref		Ref	
> 3 mg/L	96/198844	1.50(1.19,1.89)	< 0.001	1.47(1.17,1.86)	0.001
Hs-CRP (per SD)	395/1135056	1.04(0.98,1.10)	0.199	1.01(1.00,1.02)	0.222

Adjusted models were adjusted for age (10-year age classes), sex, family income, educational background, marital status, HBV infection, cirrhosis, fatty liver, BMI, TC, ALT, SUA, smoking status, drinking status, physical activity, sedentary lifestyle, tea consumption, salt intake, high-fat diet, family history of cancer

^a^ Further adjusted for hs-CRP group (≤ 3 vs. > 3)

^b^ Interaction between MetS and hs-CRP

^c^ Further adjusted for MetS

To evaluate the association between each MetS component, elevated hs-CRP levels and the development of PLC, we performed a logistic regression analysis. The results showed that WC, BP, FPG and triglycerides were all positively associated with an increase in hs-CRP levels. In addition, when all metabolic risk factors were adjusted to each other, WC, FPG and triglycerides were still positively associated with the elevated hs-CRP levels (Table S1). Table S2 showed the association between each MetS component and the PLC. Further, WC, FPG and HDL-C levels were associated with the development of PLC. After mutual adjustment, only FPG and HDL-C levels were positively associated with the incidence of PLC.

### Effect of MetS combined with hs-CRP levels on the incidence of PLC

Table 3 displays the multivariable Cox regression analysis for PLC among the four groups. The incidence densities of MetS-CRP- group, MetS-CRP + group, MetS + CRP-, and MetS + CRP + groups were 32.21, 45.36, 28.97, and 66.90/100,000, respectively. The MetS + CRP + group had the highest incidence density of PLCs. Compared with the MetS-CRP- group, the MetS + CRP + (HR = 2.91; 95% CI 1.77–4.81; *P* < 0.001) and MetS-CRP + groups (HR = 1.36; 95% CI 1.05–1.75; *P* = 0.019) were associated with higher PLC risk, but the MetS + CRP- group had no significant association with an elevated risk of PLC (HR = 1.18; 95% CI 0.76–1.82; *P* = 0.390) (Table 3).

**Table 3:** Hazard ratios (HRs) for the association of metabolic syndrome and hs-CRP levels with PLC risk

Group	Incidence density/One hundred thousand	Cases/person-years	Crude models		Adjusted models	
			HR (95%CI)	*P*-value	HR (95%CI)	*P*-value
MetS-CRP-	32.21	276/856820	Ref		Ref	
MetS-CRP +	45.36	78/171939	1.39(1.08,1.79)	0.010	1.36(1.05,1.75)	0.019
MetS + CRP-	28.97	23/79392	0.90(0.59,1.38)	0.624	1.18(0.76,1.82)	0.390
MetS + CRP +	66.90	18/26905	2.08(1.29,3.35)	0.003	2.91(1.77,4.81)	< 0.001

Adjusted models were adjusted for age (10-year age classes), sex, family income, educational background, marital status, HBV infection, cirrhosis, fatty liver, BMI, TC, ALT, SUA, smoking status, drinking status, physical activity, sedentary lifestyle, tea consumption, salt intake, high-fat diet, family history of cancer

**Fig. 2:**
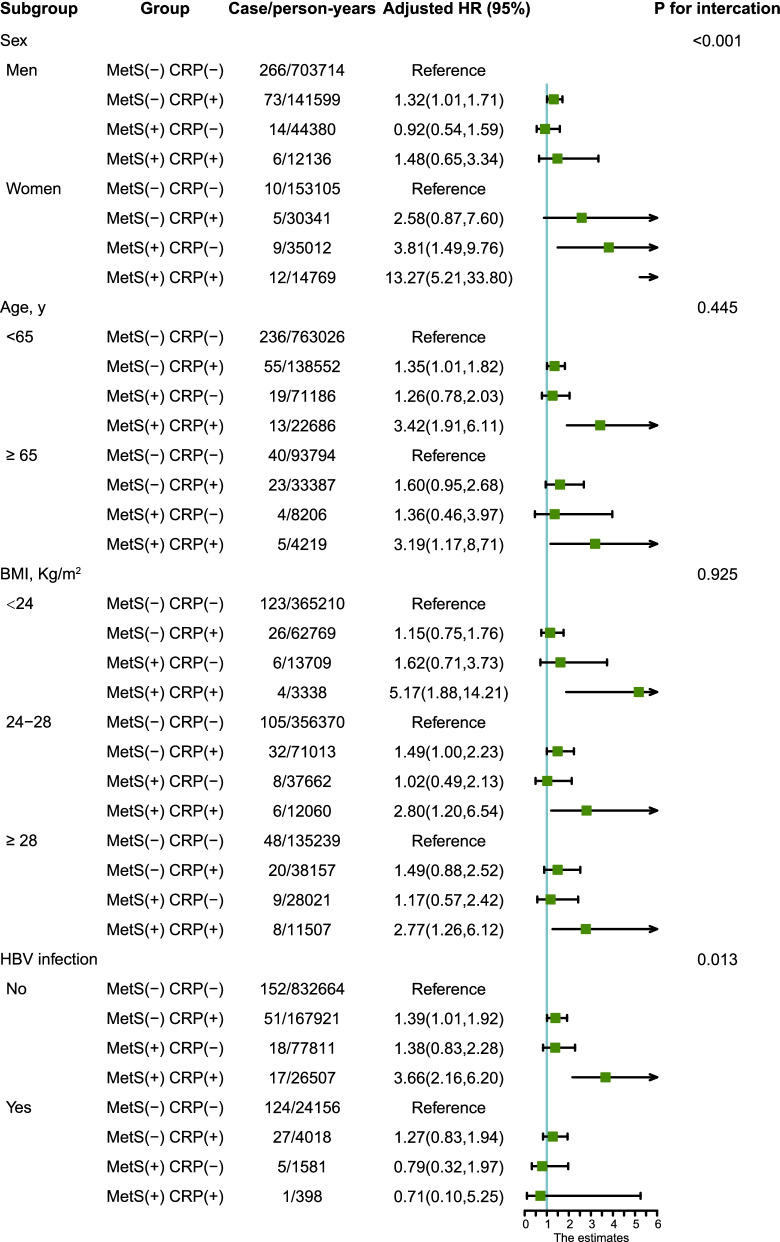
Subgroup analysis of the association between concurrence of MetS and high hs-CRP levels and PLC risk. Note: Adjusted models were adjusted for age (every 10 years), sex, family income, educational background, marital status, HBV infection, cirrhosis, fatty liver, BMI, TC, ALT, SUA, smoking status, drinking status, physical activity, sedentary lifestyle, tea consumption, salt intake, high-fat diet, family history of cancer

In the subgroup analysis (Fig. 2), similar results were observed in participants who were less than 65 years old, overweight (BMI, 24–28 kg/m^2^), or hepatitis B surface antigen (HBsAg) seronegative. Compared with the control group (MetS-CRP-), the combination of MetS and high hs-CRP levels (MetS + CRP +) was associated with a highest risk of PLC among all subgroups of BMI and age. Moreover, the co-occurrence of MetS and high hs-CRP levels presented a higher risk of PLC incidence than the control group in women participants and those who were Hepatitis B surface antigen (HBsAg) seronegative, not seropositive. In addition, co-occurrence of MetS and high hs-CRP levels and had a significant interactive effect on the risk of PLC between sexes (*P* for interaction < 0.001) and in the HBV infection subgroup (*P* for interaction = 0.012). The subgroup analysis for hs-CRP levels and MetS is shown in Figure S1. High hs-CRP levels and the sex had a significant interaction effect on the risk of PLC. Significant interaction effects on the risk of PLC were also observed between the MetS and the sex, HBV infection.

### Sensitivity analysis

To eliminate the influence of other confounding factors, we performed a sensitivity analysis by excluding the participants who were diagnosed as PLC within 1 year. The results are shown in Table S3, indicating the same trends that participants with MetS and high hs-CRP levels had the highest risk of PLC.

## Discussion

This large prospective study involving 92,770 individuals demonstrated that a combination of MetS and high hs-CRP levels was associated with an elevated risk of PLC. Further, individuals with inflammation and those without MetS had an increased risk of PLC. However, there was no significant elevation in the risk of PLC in the participants with MetS alone. As for individual MetS components, FPG and HDL-C were associated with increased risk of PLC, whereas WC, FPG and triglyceride showed significantly associated with high hs-CRP levels. This study’s findings highlight the importance of inflammation in liver cancer prevention, especially in individuals with MetS.

Over the past two decades, the number of patients with MetS has increased significantly worldwide. This increase is related to the global epidemic of obesity and diabetes. Metabolic diseases are a of the risk factor for liver cancer[18]. Both high WC and high blood glucose are associated with an increased risk of liver cancer. Low HDL cholesterol levels are also associated with an increased risk of liver cancer [19]. Metabolic syndrome (MetS) and its components have been investigated as risk factors for cancer in previous studies, but the results have been mixed. A meta-analysis of 116 datasets from 43 articles reported that the presence of MetS was associated with liver cancer (relative risk = 1.43, *P* < 0.0001). In addition, the association between MetS and cancer risk was different between ethnic groups, and a stronger association with liver cancer was found in the Asian population (*P* = 0.002) [20]. Furthermore, a previous study reported that MetS was a significant risk factor for the development of liver cancer in both sexes in the Japanese population. In a model investigating all components of the MetS, high blood glucose and low HDL-C levels were significantly associated with the incidence of liver cancer development [21]. In a large-pooled European cohort study, MetS score, along with high glucose and low cholesterol levels, was significantly associated an increased risk of PLC [22]. Based on the Surveillance, Epidemiology and End Results-Medicare-linked database (SEER-Medicare), a study showed that MetS is also a significant risk factor for the development of liver cancer in the general population of the United States of America [7].

However, not all prior studies have found that MetS increases the risk of PLC. A previous study reported that MetS is not associated with an increased risk of cancer, however, the inflammatory index was found to be an independent prognostic factor in predicting cancer incidence [23]. In our study, we found that high hs-CRP, high blood glucose, and low HDL-C levels were associated with an increased risk of PLC.

To our knowledge, our study is the first to examine whether patients with co-occurrence of high hs-CRP levels and MetS have an increased risk of PLC. We previously reported that elevated levels of hs-CRP at baseline may be associated with an increased risk of PLC [12]. CRP, as an inflammatory biomarker, has been shown to be associated with an increased risk of new-onset PLC [10, 11]. Immune response and metabolic regulation are highly integrated, and the functions of the two systems are interdependent [24]. Obesity, insulin resistance, and type 2 diabetes are closely associated with chronic inflammation, especially with the levels of CRP. Welsh et al. explored the causal relationship between obesity and inflammation using a bidirectional Mendelian randomization approach and conclude that fat mass, and obesity-related genes, and melanocortin receptor 4 single nucleotide polymorphisms lead to higher CRP levels, although there is no evidence of any reverse pathway [25]. Thus, inflammation may be a manifestation of the late-stage MetS.

The effect of the co-occurrence of inflammation and MetS on the risk of incident PLC has not been evaluated in previous studies. In our study, we found that MetS components, including WC, blood glucose, and triglycerides were significantly associated with elevated hs-CRP levels. Herein, we also systematically assessed the association between PLC risk and the co-occurrence of high hs-CRP levels and MetS, and found that co-occurrence of high hs-CRP levels and MetS strikingly increased the risk of new-onset PLC.

A previous study reported that women have a higher inflammatory overall burden than men [26]. At present, it is generally believed that sex hormones are the main factors causing the difference in inflammation levels between the sexes [27]. Obesity, especially subcutaneous adiposity, is the key correlate of CRP levels in women [28]. In addition, a study showed that elevated concentrations of hs-CRP levels were more strongly associated with MetS in women than in men [29]. In our study, Metabolic syndrome is more common in women than men. Consistently, the co-occurrence of MetS and high hs-CRP levels had a significant interactive effect on the risk of PLC with sexes in our study. The co-occurrence of MetS and high hs-CRP levels was associated with a higher risk of PLC in women than in men. Intriguingly, high hs-CRP levels or MetS had independently significant interactive effect on the risk of PLC with sexes. Thus, we concluded that the co-occurrence of high hs-CRP levels and MetS was an important risk factor of PLC in women. However, it was a challenging to determine the mechanism of this association in this study.

HBV infection is one of the main risk factors of PLC [30], especially in China. Interestingly, in our study, the proportion of participants with both high hs-CRP levels and MetS in HBsAg seropositive and cirrhotic patients were lower than those in HBsAg seronegative and non-cirrhotic patients, respectively. In addition, the co-occurrence of high hs-CPR levels and MetS was associated with an elevated risk of PLC in HBsAg seronegative participants, but not in participants with HBsAg seropositive participants. Although this is an interesting phenomenon, it is challenging to explore the mechanism of this association in this study. We speculate that this may be because patients with HBV infection are more likely to be malnourished, counteracting the effects of metabolic syndrome, or because people with HBV infection and people with liver cirrhosis pay more attention to usual physical health management and eating habits, which leads to less metabolic syndrome and inflammation in such people.

According to previous studies, there are several mechanisms that could be involved in the association between MetS and PLC: 1) Non-alcoholic fatty liver disease (NAFLD): NAFLD, the liver consequence of MetS and obesity, has a global prevalence of approximately 25% worldwide [31]. The accumulation of triglycerides in the MetS, inflammation, type 2 diabetes, and oxidative stress are the original risk factors of NAFLD [32]. These factors induce apoptosis, activate immune and inflammatory pathways, and lead to the development of fibrosis, which can progress to liver cancer. 2) Liver fibrosis: Obesity is a component of MetS. Fat cells can synthesize and release large amounts of fat proinflammatory factors and cell factors involved in insulin resistance. In addition, the lymphocytes in adipose tissue strengthen inflammation and insulin resistance, which is an important risk factor of liver fibrosis [13]. MetS and accumulation of liver lipids produce oxidative stress and lead to an increase in inflammatory factors, which are also factors of origin of liver fibrosis [33]. Further, excess lipid accumulation in liver cells can lead to the formation of lipid-filled micro and giant vesicles [34] that can lead to steatohepatitis, fibrosis, cirrhosis, and liver cancer. 3) Liver Cirrhosis: Oxidative stress and inflammation induced by MetS are also components of liver cirrhosis [35], which further develops into liver cancer. 4) Visceral obesity plays an important role in the development of MetS and is usually thought to be more easy to trigger inflammation [13], although this does occur in all patients with MetS. Moreover, the of size of the fat cells may be more important that their number. Studies have shown that large fat cells are more likely to rupture and be the focus of the apparent inflammatory cell breakdown [36]. In summary, the pro-inflammatory state is an important factor in the process of PLC. The co-existence of inflammation and MetS may be a manifestation of chronic inflammation or a high-risk form of MetS. In short, the co-existence of MetS and inflammation is a risk factor for PLC; however, the exact mechanism remains unclear. Further studies are required to confirm this association.

The strengths of our study are that it is a large prospective cohort study involving patients of a wide range of age (18–98 years old) and thus has good representativeness. In addition, the rate of loss to follow-up was nearly 0%, and the cancer diagnosis method used was reliable as it involved complete hepatitis virus infection status data collection. Nevertheless, this cohort study has several limitations. First, information on smoking, exercise, alcohol use, and medical history was self-reported and can lead to recall bias. Second, there was a lack of data on alcoholic liver disease, which may be a confounding factor. Third, the participants of Kailuan’s research were workers of the Kailuan Company, most of whom are coal miners. Therefore, the male-to-female ratio is not balanced, with males accounting for a relatively large proportion of participants. This may limit the application of extrapolation.

## Conclusion

The co-occurrence of MetS and high hs-CRP levels is associated with an increased risk of new-onset PLC in the Chinese population. Inclusion of CRP in MetS diagnostic criteria may help to identify those individuals with high-risk of PLC who should be focus population for early diagnosis and prevention of PLC in China. However, its mechanism is still unclear and needs to be verified by further study.

## Supplementary Information

**Additional file 1: Table S1.** The relationship between MetS metrics and CRP. **Table S2.** The relationship between MetS metrics and PLC. **Table S3.** Sensitivity analyses of the association of metabolic syndrome and hs-CRP levels with PLC risk. **Figure S1.** Subgroup analysis of the association between MetS or hs-CRP levels alone and PLC risk. Note: Adjusted models were adjusted for age (10-year age classes), sex, family income, educational background, marital status, HBV infection, cirrhosis, fatty liver, BMI, TC, ALT, SUA, smoking status, drinking status, physical activity, sedentary lifestyle, tea consumption, salt intake, high-fat diet, family history of cancer.

## Data Availability

The datasets generated during and/or analyses during the current study are available from the corresponding author on reasonable request.

## Abbreviations

PLC: Primary liver cancer
HBV: Hepatitis B virus
HCV: Hepatitis C virus
MetS: Metabolic syndrome
T2DM: Type 2 diabetes
hs-CRP: High-sensitivity C-reactive protein
WC: Waist circumference
BP: Blood pressure
SBP: Systolic blood pressure
DBP: Diastolic blood pressure
FPG: Fasting plasma glucose
HDL-C: High density lipoprotein cholesterol
DM: Diabetes mellitus
IQR: Interquartile range
HR: Hazard ratio
CI: Confidence interval
BMI: Body mass index
TC: Total cholesterol
ALT: Alanine transaminase
SUA: Serum uric acid
HBsAg: Hepatitis B surface antigen
