# Supplementary material for: Association between metabolic syndrome, C-reactive protein, and the risk of primary liver cancer: a large prospective study
Source: BMC Cancer. 2022 Aug 4;22:853. doi: 10.1186/s12885-022-09939-w (PMC9351132; doi:10.1186/s12885-022-09939-w)
Supplement: Supplementary file 1 — Additional file 1: Table S1. The relationship between MetS metrics and CRP. Table S2. The relationship between MetS metrics and PLC. Table S3. Sensitivity analyses of the association of metabolic syndrome and hs-CRP levels with PLC risk. Figure S1. Subgroup analysis of the association between MetS or hs-CRP levels alone and PLC risk. Note: Adjusted models were adjusted for age (10-year age classes), sex, family income, educational background, marital status, HBV infection, cirrhosis, fatty liver, BMI, TC, ALT, SUA, smoking status, drinking status, physical activity, sedentary lifestyle, tea consumption, salt intake, high-fat diet, family history of cancer. [file 12885_2022_9939_MOESM1_ESM.docx]

**Supplementary tables**

**Table S1. The relationship between MetS metrics and CRP.**

|  | Crude | |  | Adjusted | |  | Mutually adjusted | |
| --- | --- | --- | --- | --- | --- | --- | --- | --- |
|  | OR (95%CI) | *p*-value |  | OR (95%CI) | *p*-value |  | OR (95%CI) | *p*-value |
| High WC | 1.51(1.44,1.59) | <0.001 |  | 1.43(1.31,1.56) | <0.001 |  | 1.41(1.29,1.53) | <0.001 |
| High blood pressure | 1.25(1.20,1.29) | <0.001 |  | 1.05(1.01,1.08) | 0.017 |  | 1.03(0.99,1.07) | 0.099 |
| High FBG | 1.21(1.16,1.25) | <0.001 |  | 1.09(1.05,1.13) | <0.001 |  | 1.08(1.04,1.12) | <0.001 |
| High TG | 1.25(1.21,1.30) | <0.001 |  | 1.13(1.09,1.18) | <0.001 |  | 1.12(1.08,1.16) | <0.001 |
| Low HDL-C | 1.11(1.02,1.20) | <0.001 |  | 1.04(0.95,1.13) | 0.446 |  | 1.02(0.93,1.11) | 0.666 |

Note: Variables were presented as metrics of MetS.

Adjusted models were adjusted for age, sex, BMI, TC, smoking status, drinking status, physical activity. Mutually adjusted models included all MetS metrics at the same time.

**Table S2. The relationship between MetS metrics and PLC.**

|  | Crude | |  | Adjusted | |  | Mutually adjusted | |
| --- | --- | --- | --- | --- | --- | --- | --- | --- |
|  | HR (95%CI) | *p*-value |  | HR (95%CI) | *p*-value |  | HR (95%CI) | *p*-value |
| High WC | 1.22(0.98,1.52) | 0.077 |  | 1.74(1.07,2.83) | 0.025 |  | 1.61(0.99,2.61) | 0.056 |
| High blood pressure | 1.36(1.16,1.59) | <0.001 |  | 1.14(0.97,1.35) | 0.110 |  | 1.09(0.93,1.29) | 0.286 |
| High FBG | 1.58(1.35,1.86) | <0.001 |  | 1.42(1.21,1.68) | <0.001 |  | 1.39(1.18,1.63) | <0.001 |
| High TG | 1.19(1.01,1.40) | 0.035 |  | 1.13(0.96,1.34) | 0.149 |  | 1.07(0.91,2.43) | 0.423 |
| Low HDL-C | 1.26(0.90,1.76) | 0.181 |  | 1.65(1.11,2.44) | 0.013 |  | 1.64(1.11,2.43) | 0.014 |

Note: Variables were presented as metrics of MetS. Adjusted models were adjusted for age, sex, BMI, TC, hs-CRP, smoking status, drinking status, physical activity. Mutually adjusted models included all MetS metrics at the same time.

**Table S3. Sensitivity analyses of the association of metabolic syndrome and hs-CRP levels with PLC risk.**

|  | Cases/person-years | Adjusted models | |
| --- | --- | --- | --- |
|  |  | HR (95%CI) | *p*-value |
| Exclude PLC occurred within 1 year | | | |
| MetS-CRP- | 252/856807 | Ref. |  |
| MetS-CRP+ | 65/171936 | 1.24(0.94,1.64) | 0.012 |
| MetS+CRP- | 22/79391 | 1.28(0.82,2.01) | 0.276 |
| MetS+CRP+ | 17/26905 | 3.23(1.93,5.42) | <0.001 |

Note: Adjusted models were adjusted for age (10-year age classes), gender, family income, educational background, marital status, BMI, TC, ALT, SUA, smoking status, drinking status, physical activity, sedentary lifestyle, tea consumption, high-fat diet, diabetes, family history of cancer.


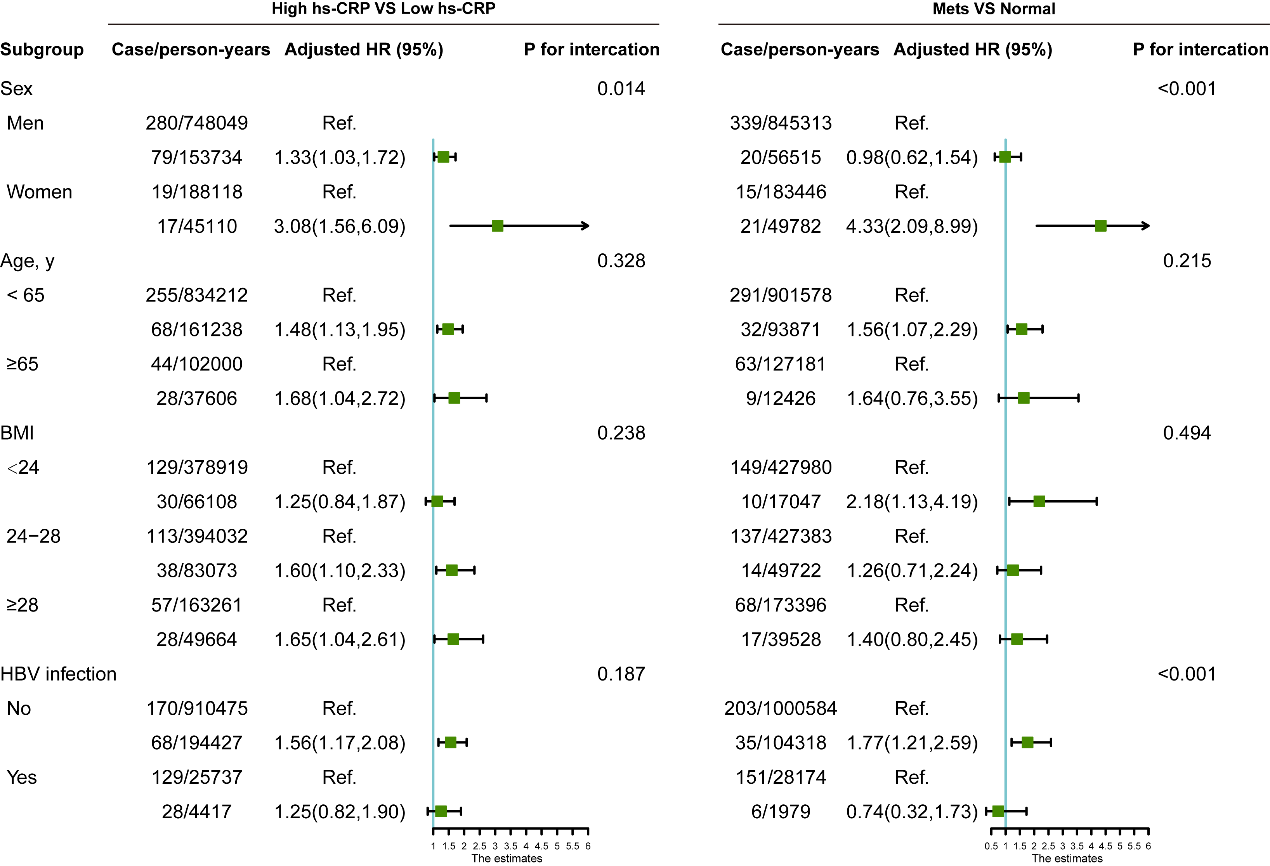


**Figure S1. Subgroup analysis of the association between MetS or hs-CRP levels alone and PLC risk.** Note: Adjusted models were adjusted for age (10-year age classes), sex, family income, educational background, marital status, HBV infection, cirrhosis, fatty liver, BMI, TC, ALT, SUA, smoking status, drinking status, physical activity, sedentary lifestyle, tea consumption, salt intake, high-fat diet, family history of cancer.
